# Supplementary material for: MgH2 nanoparticles confined in reduced graphene oxide pillared with organosilica: a novel type of hydrogen storage material
Source: Nanoscale. 2024 Jun 20;16(33):15770–81. doi: 10.1039/d4nr01524j (PMC11306994; doi:10.1039/d4nr01524j)
Supplement: NR-016-D4NR01524J-s001 [file NR-016-D4NR01524J-s001.pdf]

Electronic Supplementary Material (ESI) for Nanoscale.
This journal is © The Royal Society of Chemistry 2024

Supporting Information

MgH_2 nanoparticles confined in reduced graphene oxide pillared with organosilica: a novel type of hydrogen storage material

*Feng Yan, Estela Moreton Alfonsín, Peter Ngene, Sytze de Graaf, Oreste De Luca, Huatang Cao, Konstantinos Spyrou, Liqiang Lu, Eleni Thomou, Yutao Pei, Bart J. Kooi, Dimitrios P. Gournis, Petra E. de Jongh, and Petra Rudolf**

Dr F. Yan, E. M. Alfonsín, Dr S. de Graaf, Dr O. De Luca, Dr E. Thomou, Prof. B. J. Kooi, and Prof. P. Rudolf
Zernike Institute for Advanced Materials, University of Groningen, Nijenborgh 4, 9747 AG Groningen, the Netherlands.
E-mail: p.rudolf@rug.nl

Prof. P. Ngene, and Prof. P. E. de Jongh
Materials Chemistry and Catalysis, Debye Institute for Nanomaterials Science, Utrecht University, Universiteitsweg 99, 3584 CG Utrecht, the Netherlands.

Dr H. Cao, Dr L. Lu, and Prof. Y. Pei
Engineering and Technology Institute Groningen, University of Groningen, Nijenborgh 4, 9747AG Groningen, the Netherlands.

Dr K. Spyrou, Dr E. Thomou, and Prof. D. P. Gournis
Department of Materials Science and Engineering, University of Ioannina, 45110 Ioannina, Greece.

Synthesis of rGO-BTB heterostructure

The layered heterostructure of reduced graphene oxide and organosilica was prepared with a soft-template method. The synthesis of graphene oxide (GO) from graphite flakes was based on a modified Staudenmaier's method, as described in our previous publications.[1,2] A suspension of 2.5 mg mL^{-1} of GO in water was adjusted to the pH of 7.5 by adding small amounts of 0.1 M NaOH solution while monitoring with a pH meter (Mettler Toledo, FP20-Meter); the color of suspension changed from brownish yellow to dark brown. A surfactant, dodecylamine, was mixed with the GO suspension in a weight ratio of 3.0 (dodecylamine/GO) for a prepillaring step, which ensures that the graphene oxide interlayer space is sufficiently opened. After stirring for 24 h, the dodecylamine-intercalated graphene oxide was separated by centrifugation at 4500 rpm and washed three times with ethanol/water (v/v = 1:1) to remove the excess dodecylamine, before being vacuum dried at room temperature. For the intercalation of the phenylene-bridged organosilica precursor into the interlayer space of GO, 100 mg of dodecylamine-intercalated GO were dispersed in 5 mL n-butanol, and the organosilica precursor BTB was added dropwise to the solution while vigorously stirring for 2 h. Then water was added while keeping the molar ratio of silica precursor/H_2O/n-butanol constant at 1/4/54. The sol-gel reaction was performed at 50 ^{o}C by stirring for 2 h. The obtained gel was placed in the oven at 50 °C and dried overnight. The layered heterostructure of reduced graphene oxide and organosilica (rGO-BTB) was obtained by removing the amine-surfactant with a calcination step of 370 °C in air for 2 h, with the heating rate of 1.0 °C min^{-1}.

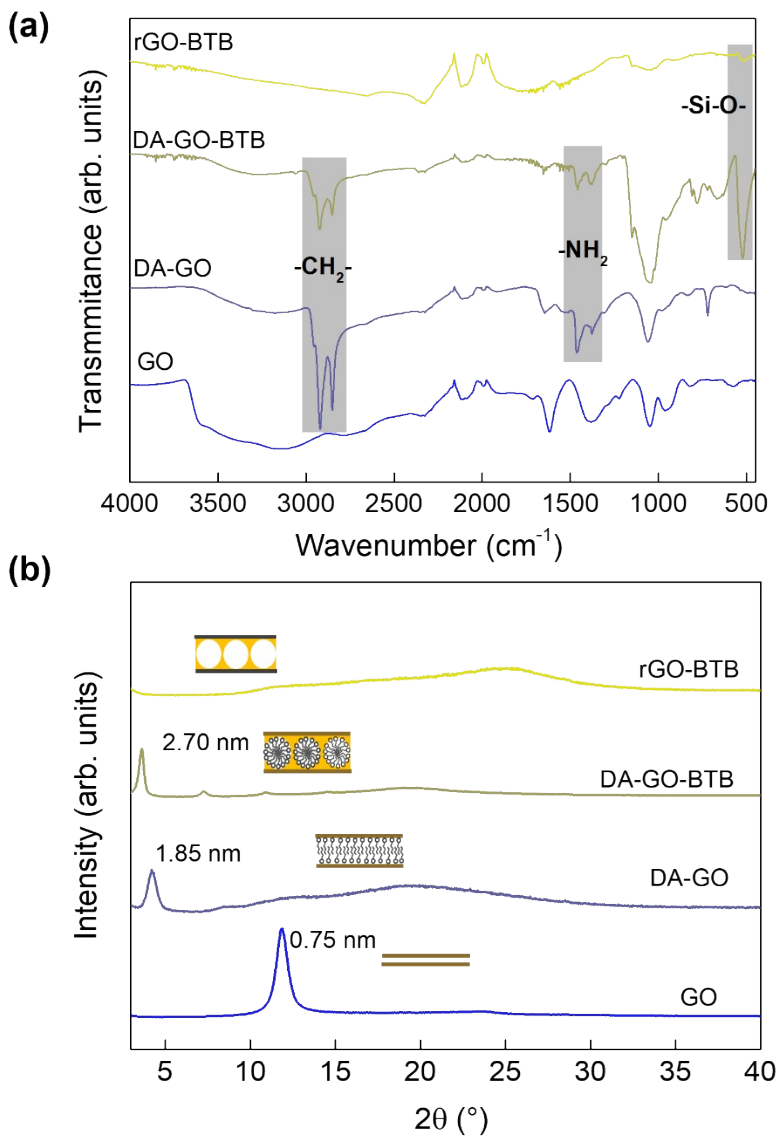

Figure S1. (a) FTIR spectra of GO, dodecylamine-intercalated GO (DA-GO), GO intercalated with both dodecylamine and BTB (DA-GO-BTB) and rGO-BTB; (b) X-ray diffraction patterns of GO, GO intercalated with dodecylamine (DA-GO), GO with dodecylamine and BTB (DA-GO-BTB) and rGO-BTB.

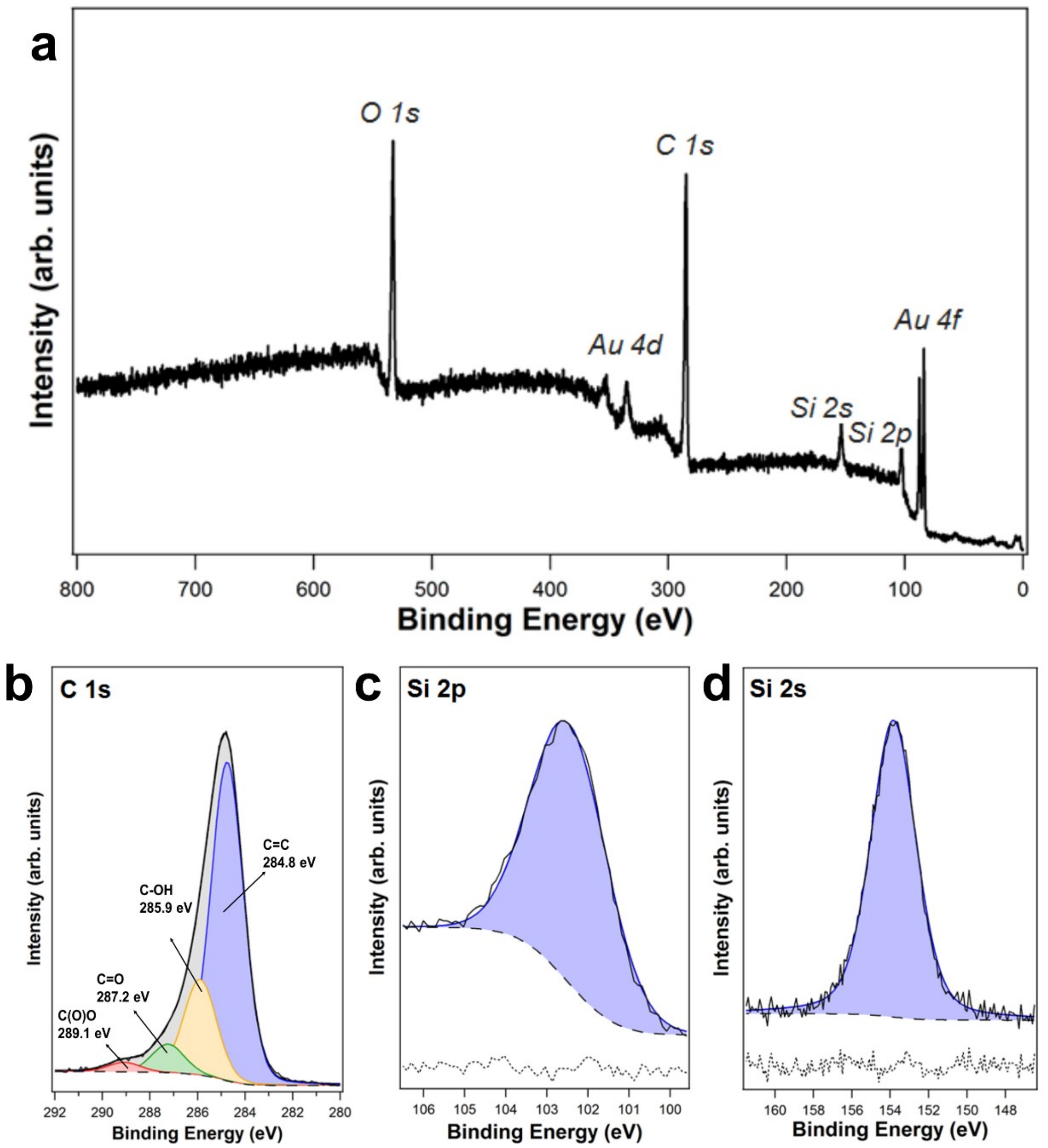

Figure S2. X-ray photoelectron spectra of rGO-BTB (a) wide scan, (b) C1*s*, (c) Si2*p* and (d) Si2*s* core level regions.

Table 1 Specific surface area, pore volume of rGO-BTB, and of the MgH_2/rGO-BTB-10 and MgH_2/rGO-BTB-20 composites

Samples	specific surface area (SSA) [$m^2\ g^{-1}$]			pore volume [$cm^3\ g^{-1}$]		
	S_{total}	$S_{t\text{-}plot\ micro}$	$S_{t\text{-}plot\ external}$	V_{total}	V_{micro}	V_{meso}
rGO-BTB	342	236	76	0.145	0.091	0.054
MgH_2/rGO-BTB-10	212	147	65	0.096	0.072	0.024
MgH_2/rGO-BTB-20	75	24	51	0.093	0.012	0.081

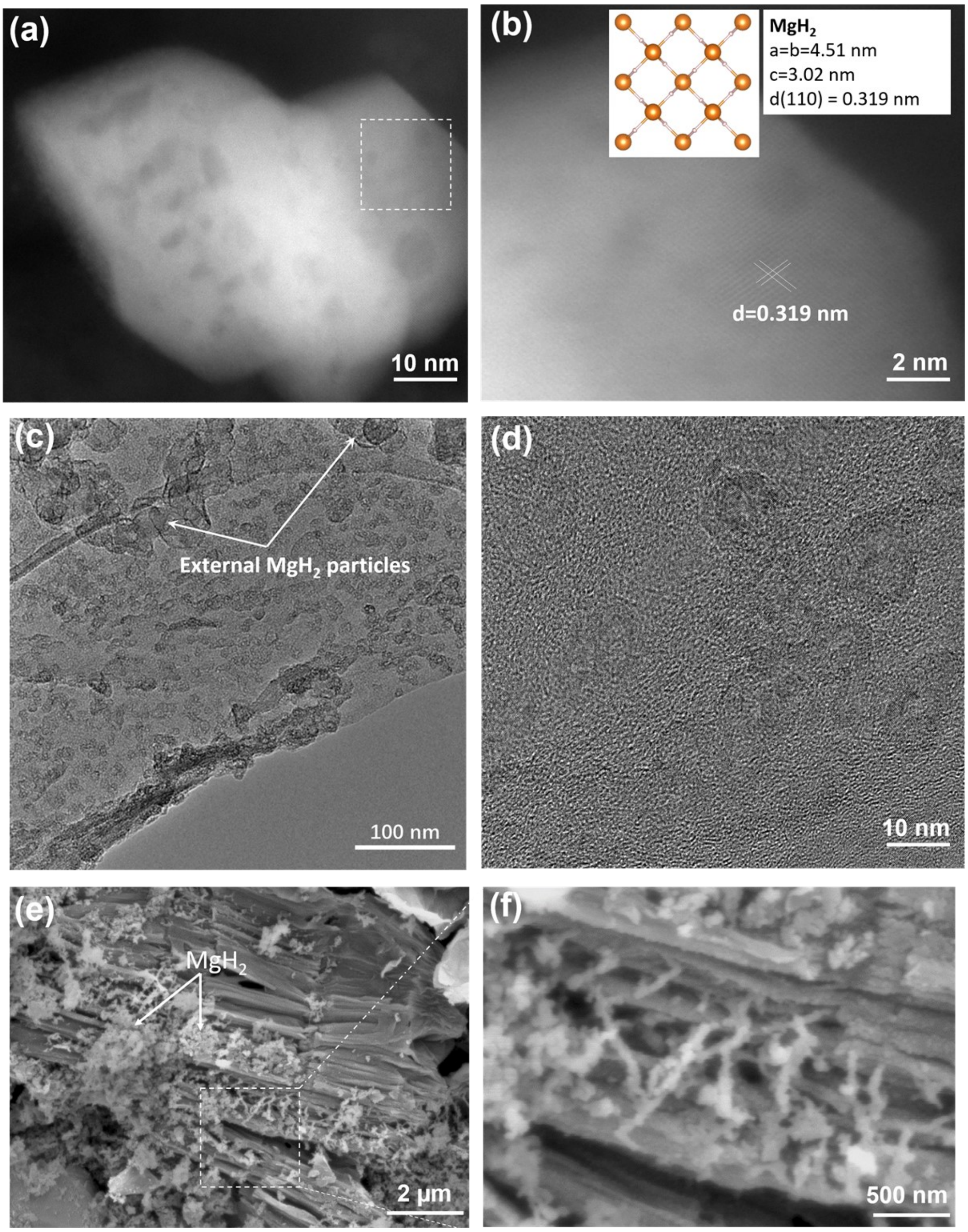

Figure S3. (a) Residual MgH_2 particles located outside of rGO-BTB heterostructure of MgH_2/rGO-BTB-20; (b) enlargement of the area marked with a dashed white line in (a), inset: structure of crystalline MgH_2; (c) and (d) TEM images of MgH_2/rGO-BTB-20; (e) and (f) SEM images of MgH_2/rGO-BTB-20.

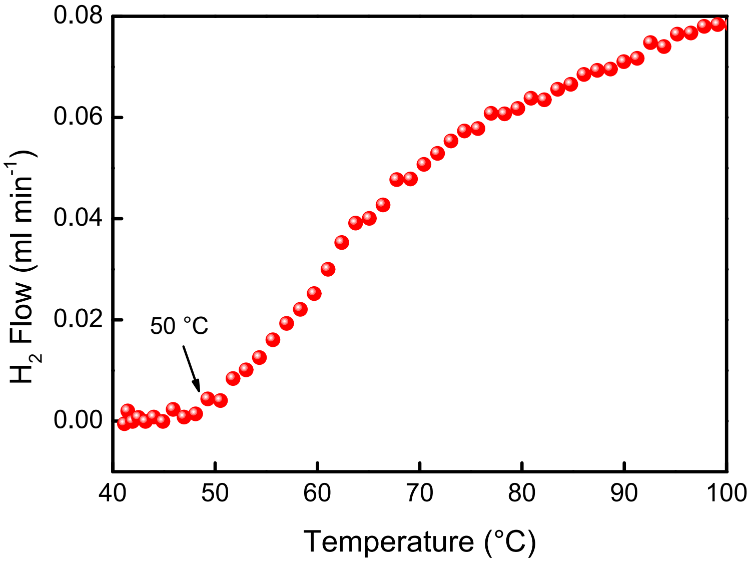

Figure S4 Temperature programmed desorption (TPD) spectrum of MgH_2/rGO-BTB-10 in the region of 40-100 °C recorded with a heating rate of 5 °C min^{-1}.

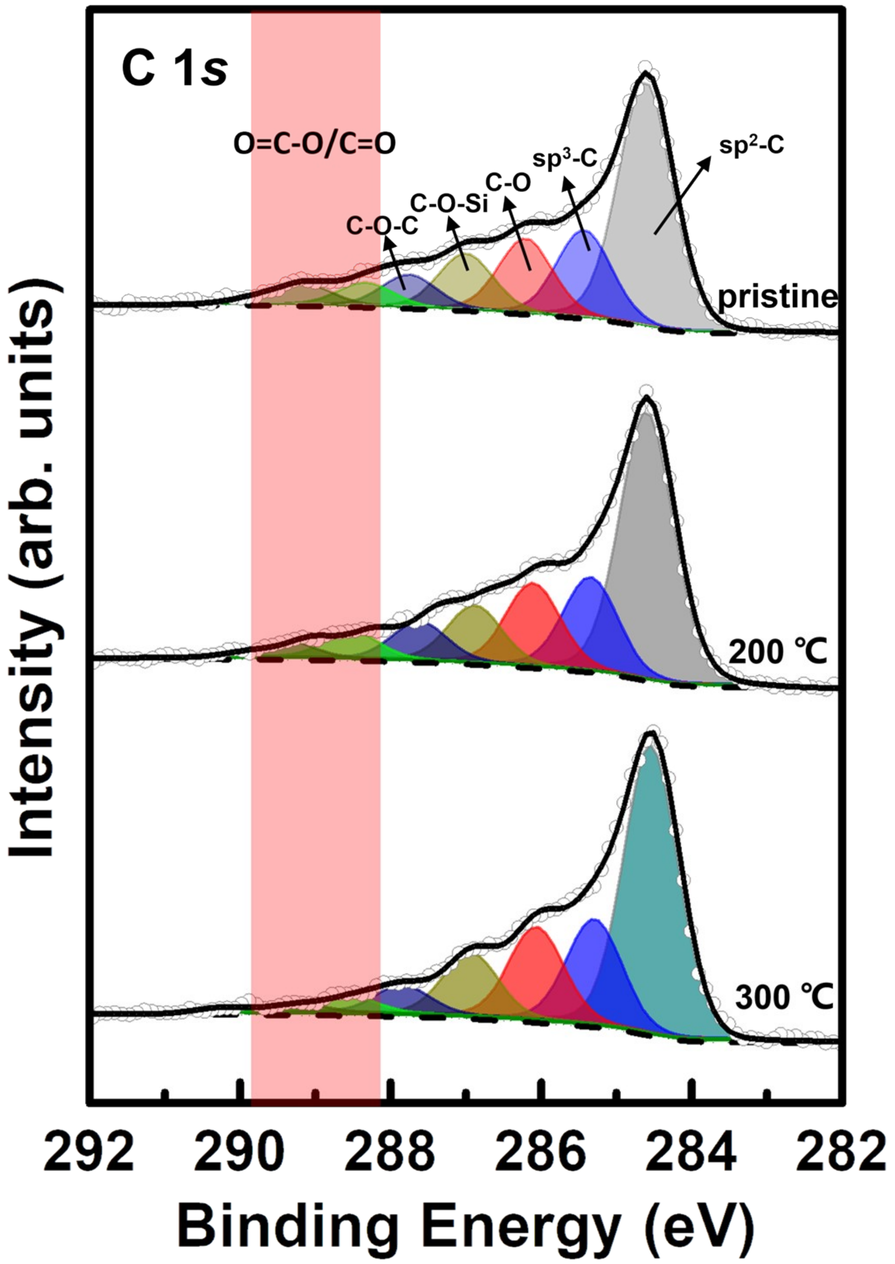

Figure S5. XPS spectra of the C1*s* core level region of MgH_2/rGO-BTB-10 recorded on the pristine material and after heating to 200 and to 300 °C.

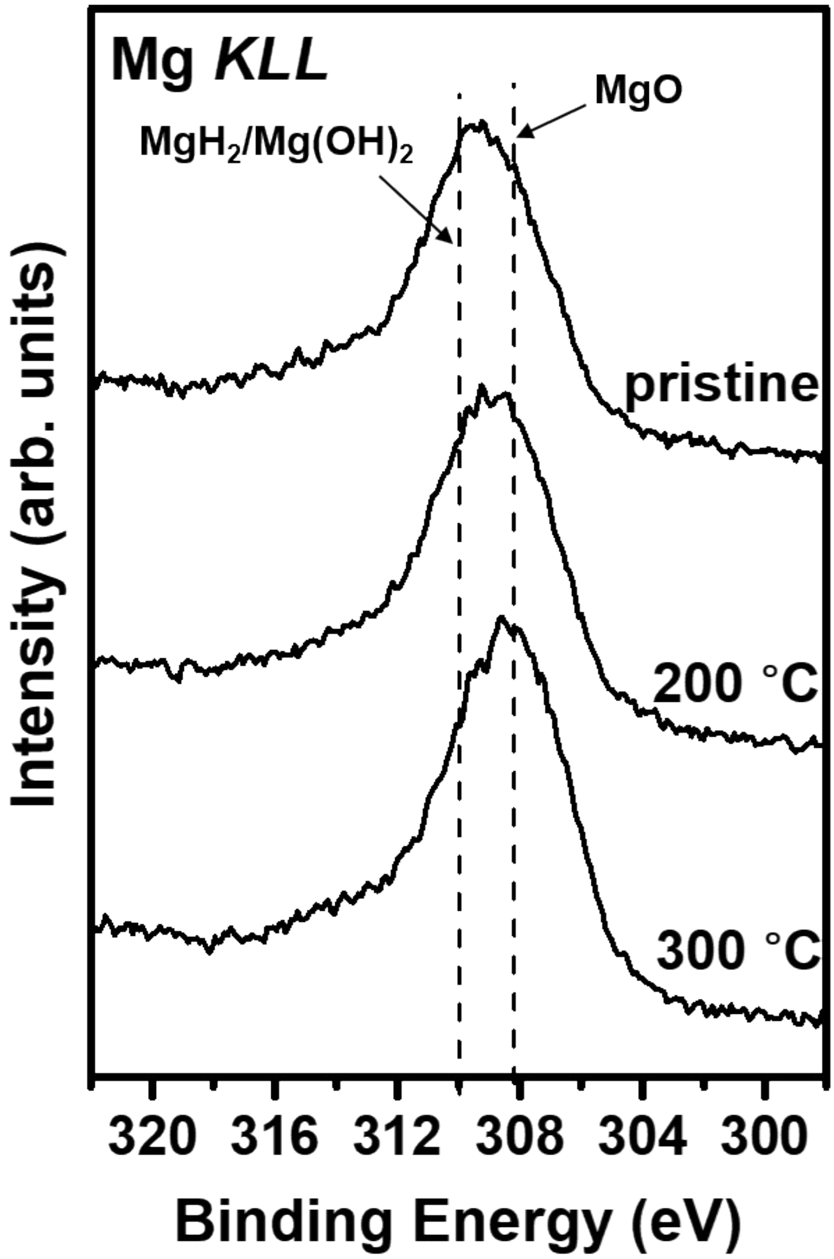

Figure S6. The Mg *KLL* Auger region of the XPS spectra of MgH_2/rGO-BTB-10 recorded before and after heating to 200 and to 300 °C.

Table S1. Binding energies and intensity ratios of C1*s* core level components as deduced from the XPS spectra collected MgH_2/rGO-BTB-10 recorded before and after heating to 200 and to 300 °C.

species	pristine	200 ℃	300 ℃
Sp^2-C	284.7 eV (44.9%)	284.6 eV (46.0%)	284.5 eV (47.4%)
Sp^3-C	285.4 eV (16.0%)	285.3 eV (16.0%)	285.3eV (17.2%)
C-OH	286.3 eV (13.6%)	286.1 eV (14.2%)	286.0eV (15.5)
C-O-Si	287.1 eV (10.3%)	286.9 eV (9.7%)	286.9 eV (10.3%)
C-O-C	287.8 eV (6.0%)	287.6 eV (6.6%)	287.8 eV (4.6%)
C=O	288.5 eV (4.5%)	288.4 eV (4.6%)	288.5eV (2.9%)
O-C=O	289.2 eV (4.2%)	289.2 eV (2.9%)	289.3 eV (2.0%)

References

1. F. Yan, S. Kumar, K. Spyrou, A. Syari'ati, O. De Luca, E. Thomou,; E. M. Alfonsín, D. Gournis, P. Rudolf, *ACS ES&T Water* **2021**, *1*, 157.

2. K. Spyrou, M. Calvaresi, E. K. Diamanti, T. Tsoufis, D. Gournis, P. Rudolf, F. Zerbetto, *Adv. Funct. Mater.* **2015**, *25*, 263.
